# Extended Functional Groups (EFG): An Efficient Set for Chemical Characterization and Structure-Activity Relationship Studies of Chemical Compounds

**DOI:** 10.3390/molecules21010001

**Published:** 2015-12-23

**Authors:** Elena S. Salmina, Norbert Haider, Igor V. Tetko

**Affiliations:** 1 Institute for Organic Chemistry, Technical University Bergakademie Freiberg, Leipziger Str. 29, Freiberg D-09596, Germany; salmina@mailserver.tu-freiberg.de; 2 Department of Pharmaceutical Chemistry, University of Vienna, Althanstraße 14, Vienna A-1090, Austria; norbert.haider@univie.ac.at; 3 Institute of Structural Biology, Helmholtz Zentrum München—Research Center for Environmental Health (GmbH), Ingolstädter Landstraße 1, b. 60w, Neuherberg D-85764, Germany; 4 BigChem GmbH, Ingolstädter Landstraße 1, b. 60w, Neuherberg D-85764, Germany

**Keywords:** chemical functional groups, heterocyclic compounds, chemoinformatics analysis, machine learning, data interpretation

## Abstract

The article describes a classification system termed “extended functional groups” (EFG), which are an extension of a set previously used by the CheckMol software, that covers in addition heterocyclic compound classes and periodic table groups. The functional groups are defined as SMARTS patterns and are available as part of the ToxAlerts tool (http://ochem.eu/alerts) of the On-line CHEmical database and Modeling (OCHEM) environment platform. The article describes the motivation and the main ideas behind this extension and demonstrates that EFG can be efficiently used to develop and interpret structure-activity relationship models.

## 1. Introduction

Chemical functional groups are an important concept in chemistry. As defined by the IUPAC Goldbook “The functional group is an atom, or a group of atoms that has similar chemical properties whenever it occurs in different compounds. It defines the characteristic physical and chemical properties of families of organic compounds” [1]. The functional group is the basis for the naming of chemical compounds in the IUPAC systematic nomenclature. In principle, under the assumption that some properties are in general additive with respect to functional groups, the property of a new molecule can be easily calculated as a sum of properties of individual functional groups and their interactions. This idea is heart of the UNIversal quasichemical Functional group Activity Coefficients (UNIFAC) method [2] which is used for the prediction of activity coefficients in nonelectrolyte liquid mixtures. The chemical functional groups play also a very important role for understanding of chemical reactions by providing an important abstraction level for their categorization and analysis.

In contrast to functional chemical groups, which are derived based on chemical expert knowledge, it is also possible to generate structural groups in a completely automated manner based on some pre-defined rules. The examples include Isida fragment descriptors [3], MolPrint [4] and ECFP [5] fingerprints as well as many other which use different ways of subdividing the chemical structures into fragments based on some mathematical algorithms. Such generated groups are frequently used with machine learning methods, to analyze diversity of chemical libraries, *etc.* However, the groups generated in this way are in general strongly overlapping (many atoms belong to different groups) and are more difficult to interpret.

The development of Quantitative Structure Activity/Property Relationship (QSAR/QSPR) models can greatly benefit from using interpretable and easy to calculate descriptors. The chemically relevant functional chemical groups in this respect on one side can contribute to a model with high prediction ability while on the other side can provide a more easy interpretation of the detected relationships.

While there is a large number of software tools for the automatic calculation of chemical groups, only a few open access tools can be used for chemical functional characterization. One of the first tools, the CheckMol program [6], which incorporated 200 functional groups, was released in 2005. Recently, several new developments in this field were initiated. A set of generic chemical functional groups was made available as a part of the ToxPrint set of chemotypes [7]. Wishart’s lab has developed the ClassyFire taxonomy, which covers 4820 chemical classes of organic and inorganic compounds. This service is publicly available and can be queried on-line using the REST interface (http://classyfire.wishartlab.com).

Based on such sets of well-defined chemical functionalities, controlled vocabularies (ontologies) can be defined which help to classify small organic molecules in chemical space [8]. Fingerprints derived from functionality patterns have also been successfully employed for a machine-learning approach to predict whether an organic molecule is a substrate/non-substrate or an inhibitor/non-inhibitor of the P-glycoprotein transporter system, an essential aspect for multi-drug resistance observed e.g., in tumor chemotherapy [9]. Other applications include the use of automatically generated binary patterns representing the functional groups in a molecule as an extremely fast search criterion in structure and reaction databases [10].

In this article we report a new set of 583 manually curated extended functional groups (EFG) which was developed based on our earlier set of about 200 groups [10] available in the CheckMol program [6]. We describe the logic used to extend the previous set and introduce new groups, as well as exemplify their use for QSAR/QSPR modeling and interpretation of functional features separating mutagenic and non-mutagenic compounds for Ames toxicity.

## 2. Results and Discussion

Below, we show two examples of a use of the EFG for data modeling and analysis of overrepresented structures in compounds, which are active in the Ames test. In these studies, we will refer to functional groups defined in our previous publication [10] as CheckMol-FG and to the set of extended functional groups as EFG.

### 2.1. Functional Groups as Descriptors

The counts of functional groups in molecules can be used as descriptors for the development of models. The use of functional groups defined in CheckMol-FG resulted in models with much lower accuracy compared to the models developed with the top-performing sets of descriptors for both regression (Table 1) and classification (Table 2) tasks. For this analysis we selected 20 datasets, which covered different properties ranging from simple physico-chemical (water solubility [11], melting [12] and pyrolysis [13] points) to complex biological properties (lowest effect level [14], estrogen receptor binding [15]) as well as included an example of analysis of chemical mixtures [16]. In particular, for small datasets the accuracy of models developed with EFG was similar to that of the best models developed with other descriptor sets using the same methods. The models developed with the EFG set had higher performance compared to that of models developed with CheckMol-FG with an exception of classification of azeotropes (Table 2). However, for azeotropes the EFG based model calculated significantly higher balanced accuracy (82% ± 4% *vs.* 74% ± 4%) for the test compounds. Thus, the difference in prediction performances for the training set could be statistical variation due to small dataset size. Thus, the extension of CheckMol-FG dramatically increased the accuracy of models developed using the new set of EFG descriptors.

**Table 1:** Comparison of the performance of regression models developed using functional groups with previously published ones.

Property	Original Models					Based on CheckMol-FG		New EFG Descriptors	
	Best descriptors ^a^	N	Method	RMSE	R^2^	RMSE	R^2^	RMSE	R^2^
Environmental toxicity against *T. Pyriformis* [17]	Estate	644	ASNN	0.44 ± 0.02	0.83 ± 0.02	0.8 ± 0.03	0.44 ± 0.04	0.48 ± 0.03	0.8 ± 0.02
logP for Pt(II/IV) complexes [18]	Fragmentor (out of 12)	233	ASNN	0.43± 0.03	0.92± 0.02	1.42 ± 0.07	0.16 ± 0.05	0.45± 0.03	0.91± 0.02
HIV inhibition [19]	Dragon (out of 10)	286	ASNN	0.48± 0.03	0.87± 0.02	0.68 ± 0.03	0.75 ± 0.03	0.55 ± 0.03	0.83 ± 0.02
Melting point [12]	CDK (out of 10)	47427	ASNN	39.1 ± 0.2	0.76 ± 0.01	50.6 ± 0.2	0.59 ± 0.01	45.1 ± 0.2	0.67 ± 0.01
Melting point [13]	Fragmentor (out of 12)	275133	LibSVM	35.4 ± 0.1	0.69 ± 0.01	47.5 ± 0.1	0.46 ± 0.01	40.5 ± 0.1	0.61 ± 0.01
Lowest Effect Level (LEL) toxicity prediction challenge [14,20]	Adriana (out of 10)	483	ASNN	0.93 ± 0.03	0.22 ± 0.04	0.98 ± 0.05	0.16 ± 0.04	0.97 ± 0.05	0.17 ± 0.04
Solubility in water [11]	Estate	1311	ASNN	0.62 ± 0.2	0.91 ± 0.01	1.25 ± 0.04	0.65 ± 0.02	0.66 ± 0.02	0.90 ± 0.01
Pyrolysis point [13]	Estate	13769	LibSVM	35.6 ± 0.2	0.55 ± 0.01	42.1 ± 0.3	0.38 ± 0.01	38.7 ± 0.2	0.47 ± 0.01
PTB1B inhibition [21]	Dragon	2237	ASNN	0.77 ± 0.02	0.71 ± 0.02	0.96 ± 0.02	0.55 ± 0.02	0.81 ± 0.02	0.68 ± 0.02
Estrogen Receptor binding [15]	ALOGPS + Estate	1677	ASNN	0.062 ± 0.004	0.58 ± 0.06	0.084 ± 0.006	0.33 ± 0.04	0.079 ± 0.006	0.34 ± 0.04

^a^ The descriptors (the abbreviations are explained in the respective articles), which provided the best performance of models developed for the respective training set as well as number of the analyzed descriptor sets are indicated. RMSE—Root Mean Squared Error; R^2^ is square of Pearson linear correlation coefficient; ASNN is Associative Neural Network [22]; LibSVM is Support Vector Machine [23].

**Table 2:** Comparison of the performance of classification models developed using functional groups with previously published ones.

Property	Original Models					CheckMol-FG Descriptors		EFG Descriptors	
	Best descriptors ^a^	N	Method	BA	MCC	BA	MCC	BA	MCC
AMES test [24]	Estate	4361	ASNN	77.5% ± 0.6%	0.55 ± 0.01	74.4% ± 0.6%	0.49 ± 0.01	76.2% ± 0.6%	0.53 ± 0.01
Ready biodegradability [25]	CDK (out of 7)	1884	ASNN	86.7% ± 0.8%	0.72 ± 0.02	75% ± 1%	0.49 ± 0.02	83.2%± 0.9%	0.65 ± 0.02
Solubility in DMSO [26]	ALOGPS + Estate (out of 9)	50620	ASNN	73.8% ± 0.4%	0.24 ± 0.01	57.1% ± 0.4%	0.07 ± 0.01	71.5% ± 0.5%	0.22 ± 0.01
CYP450 inhibition [27]	Dragon (out of 10)	3737	J48	82.1% ± 0.6%	0.64 ± 0.01	78.9% ± 0.7%	0.59 ± 0.01	79.5% ± 0.7%	0.59 ± 0.01
Pyrolysis/ Melting point classification [13]	Estate (out of 10)	241699	LibSVM	78.2%± 0.2%	0.33 ± 0.01	53.1% ± 0.2%	0.04 ± 0.01	74.2%± 0.2%	0.27 ± 0.01
Androgen receptor binding [28]	Dragon	744	ASNN	77% ± 2%	0.54 ± 0.03	70% ± 2%	0.41 ± 0.04	77% ± 2%	0.54 ± 0.03
Ransthyretin receptor binding [28]	Dragon	162	ASNN	89% ± 3.0%	0.79 ± 0.05	83% ± 3%	0.67 ± 0.06	86% ± 3%	0.72 ± 0.06
Estrogen Receptor binding [15]	Dragon (out of 11)	1677	ASNN	74% ± 2.0%	0.39 ± 0.03	62% ± 2%	0.33 ± 0.04	72% ± 2%	0.34 ± 0.03
Azeotropes classification [16]	Adriana	465	RF	78% ± 2%	0.55 ± 0.04	77% ± 2%	0.55 ± 0.04	75% ± 2%	0.49 ± 0.04
ATAD5 genotoxicity [29]	Dragon	9363	ASNN	78% ± 1%	0.28 ± 0.01	58% ± 1%	0.07 ± 0.01	74% ± 1%	0.22 ± 0.01

^a^ See footnotes of Table 1. BA is Balanced Accuracy; MCC is Matthews correlation coefficient; RF is random Forest [30]; J48 is Weka [31] implementation of decision trees.

### 2.2. Analysis of Datasets

**Figure 1:**
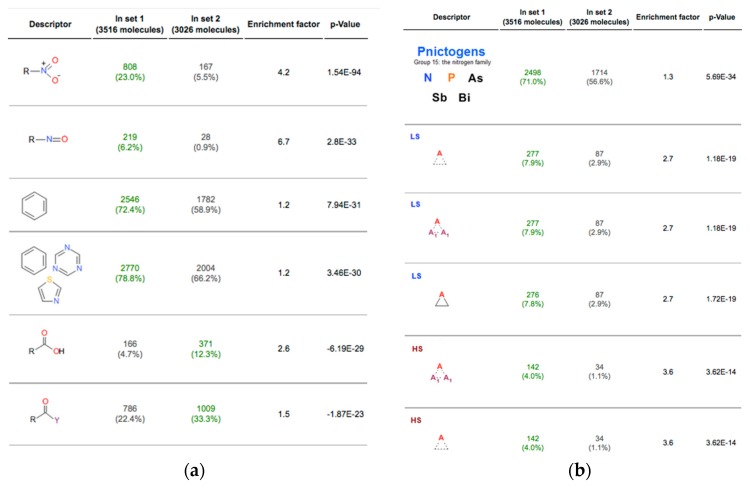
Examples of overrepresented functional groups detected using (**a**) initial set of CheckMol-FG functional groups [6,10] and (**b**) the extended EFG set. *p*-values are calculated using hyper-geometric distribution. Only six most significant groups are shown for each set of descriptors. The full list of significant groups is available at http://ochem.eu/article/27542.

The analysis of overrepresented functional groups can allow better elucidation of differences between both sets. The functional groups have been already successfully used for this purpose in the previous studies. For example, the functional groups have been used to identify some groups’ contribution to biodegradability of chemical compounds or their propensity to be soluble in DMSO [26]. Figure 1 illustrates overrepresented groups for Ames active and inactive molecules [24] using the SetCompare utility of OCHEM. The use of the initial CheckMol definition detected several significantly overrepresented groups, such as nitro, nitroso or azides, which strongly contribute to the mutagenicity of molecules. Contrary to that, carboxylic acids or their derivatives, aryl chlorides or in general aryl halides apparently decrease the mutagenic properties.

The use of EFG doubled the number of significant groups as compared to the use of the old algorithm. Importantly, the newly used groups are more general and allow better generalization of chemical structures. For example, the most significant overrepresented group in Ames positive compounds is the class of Pnictogens, thus indicating a propensity of nitrogen family compounds to cause mutagenicity. The various three-membered heterocycles (especially, aziridines) that are known mutagenic agents [32] were also overrepresented among the mutagenic compounds. Contrary to that, a saturated four-membered heterocycles with one fusion heteroatom is a commonly met fragment among non-mutagenic compounds what indicates that this group is likely not responsible for mutagenicity. These functional groups were not defined in the original definition and were part of “aromatic heterocyclic” and “heterocyclic” compounds with lower *p*-values. The use of EFG allows better highlighting the higher mutagenic propensity of smallest sub-structures of this class. It needs to be mentioned that mutagenicity as well as the other types of adverse effects can be provoked by the presence of two (or more) EFGs and the additional analysis might help to identify such co-effect.

The use of SMARTS patterns for the definition of EFG sets offers ample flexibility to re-adjust and fine-tune these descriptors depending on the need of an individual project. The nitro-groups are known to strongly correlate with Ames mutagenicity [24,32]. Indeed, the nitro-groups were highly overrepresented among mutagenic compounds (23%) as compared to non-mutagenic ones (5.5%, enrichment factor 4.2). Based on the general nitro-group SMARTS, we developed two additional patterns to better characterize the structural environment of nitro groups, namely nitro-groups connected to aromatic (“aromatic nitro-groups”) and aliphatic (“aliphatic nitro-groups”) moieties. The enrichment factor, 4.6, for the aromatic nitro-group was higher than that observed for the general nitro-group (4.2), thus indicating a higher mutagenic propensity of nitro-groups connected to an aromatic moiety. Contrary to that, the compounds with a non-aromatic nitro group were equally distributed between mutagenic and non-mutagenic compounds. This result provides a testable hypothesis that nitro-groups connected to a non-aromatic moiety are non-mutagenic.

## 3. Methods

### 3.1. Extension of Functional Groups (FG) Recognized by the CheckMol

**Figure 2:**
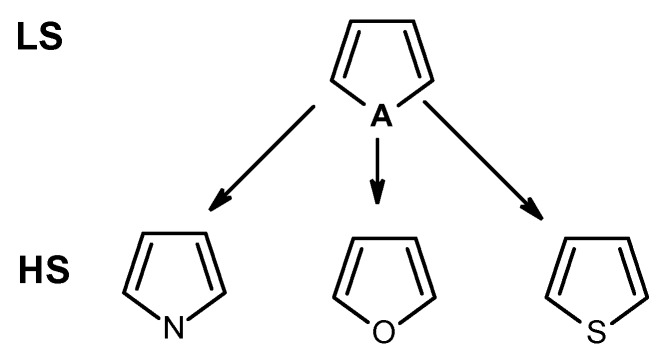
Examples of high specificity (HS) and low specificity (LS) functional group patterns for recognizing aromatic 5-membered heterocycles with one heteroatom. “A” denotes any “aromatic” atom except carbon.

As the basis set for our development, we used the functional groups (FG) recognized by the CheckMol program [6]. All CheckMol-FG were encoded using SMARTS and verified against the original program. This list was augmented with FG known to be responsible for compound reactivity or propensity to a metabolic transformation (e.g., vinyl, alkynyl and allyl halides, alcohols and thiols; amino(thio)phenols; quinones) [33]. A sizeable portion of the developed EFG was devoted to heterocyclic compounds. Heterocyclic rings are actively used in developing biologically active compounds, high-energy and conductive materials, *etc.*, so the respective grouping might be of interest for specialists from different areas [34,35]. Indeed, the comprehensive description of these classes of compounds could be important for better characterization of their chemical properties and biological activity. High specificity (HS) and low specificity (LS) functional group patterns were developed for describing heterocycles. For example, aromatic five-membered heterocycles with one heteroatom are considered as part of a LS-group pattern while pyrrole, furan and thiophene rings form HS-group patterns (Figure 2). The HS-patterns do not allow fusion between cycles while it is tolerated for LS-patterns. Thus, the HS patterns only match chemicals that include the exact heterocyclic moiety in question while LS provide a generalization of such groups.

Additionally, a set of patterns for compounds containing atoms from one or another periodic table group or category such as non-metals, metalloids, transition metals *etc.* was added (see Supplementary Materials for more details). Such classification might be necessary during the study of coordination complexes or for tasks requiring recognition of compounds containing a specific set of atoms.

### 3.2. Technical Implementation

The functional groups were implemented as SMARTS strings and are available under http://ochem.eu/alerts. The ChemAxon SMARTS search engine is used to identify and count the number of occurrences of the group’s chemical structure in molecules. Special care is taken to have consistent results across various chemical representations of chemical structures such us definitions of aromaticity. To avoid this dependency, each structure is first converted into its Kekulé representation and after that aromatized using the ChemAxon software. A listing of structures and names of the selected functional groups is provided as Supplementary Materials to this article.

## 4. Conclusions

We have developed an extended set of functional groups (EFG), which was augmented with chemical functional groups (FGs), which are relevant for medicinal chemistry as well as providing better coverage of heterocyclic compounds.

Our approach is very different to the generation of arbitrary SMARTS, which can produce myriads of patterns. The developed EFGs are well known chemical functional groups, namely molecular fragments that are responsible for chemical properties of compounds. Their usage, as opposed to ANY SMARTS, allows analyzing gained results in a very effective way, providing a mechanistically based interpretation. EFG are not overlapping (except for high and low specificity patterns). Moreover, the development efforts were to describe heterocycles, which are of utmost interest to medicinal chemistry.

The EFGs almost tripled the number of FGs from the 200 originally used in the CheckMol program to 583. This extension significantly improved the prediction ability of machine learning models developed with EGF descriptors, as compared to those developed using the initial set of descriptors. Moreover, as exemplified for analysis of compounds active in the Ames test, EFG better elucidate which compound features contribute to the toxicity of chemicals.

It should be mentioned that the current version of EFG is not “carved in stone”, but offers enough flexibility for further fine-tuning. The use of SMARTS patterns for the definition of EFG sets offers ample flexibility to re-adjust and fine-tune these descriptors depending on the demand of individual projects. The envisaged optimizations should be facilitated by the constantly growing availability of biological data. The public availability of the EFG groups on the OCHEM web site will contribute to their wider use for the analysis and interpretation of chemical and biological properties of chemical compounds [36].

## Supplementary Materials

Table of group names of extended functional groups (EFGs) are available as part of the ToxAlerts tool (http://ochem.eu/alerts) and can be accessed at: http://www.mdpi.com/1420-3049/21/1/1/s1.
