# Supplementary material for: Extended Functional Groups (EFG): An Efficient Set for Chemical Characterization and Structure-Activity Relationship Studies of Chemical Compounds
Source: Molecules. 2015 Dec 23;21(1):1. doi: 10.3390/molecules21010001 (PMC6273096; doi:10.3390/molecules21010001)
Supplement: Supplementary file 1 [file molecules-21-00001-s001.pdf]

Supplementary Materials: Extended Functional Groups (EFG): An Efficient Set for Chemical Characterization and Structure-Activity Relationship Studies of Chemical Compounds

Elena S. Salmina [1], Norbert Haider [2] and Igor V. Tetko [3,4,*]

Group names of extended functional groups (EFGs) available as part of the ToxAlerts tool (http://ochem.eu/alerts).

Visual depictions of the corresponding EFG can be found online in ToxAlerts tool. LS and HS correspondingly denote low and high specificity functional group patterns (for the explanation please refer to the main article or ToxAlerts tool).

Chemistry of the Elements

- Alkali metals
- Alkaline earth metals
- Transition metals
- Lanthanoids
- Actinoids
- Post-transition metals
- Metalloids
- Nonmetals

- Tetragens (carbon group)
- Pnictogens (nitrogen group)
- Chalcogens (oxygen group)
- Halogens
- Noble gases

- Organic and inorganic cations and anions
  - Any cations
  - Any anions

Chemistry of ORGANIC Compounds

Hydrocarbons

- Alkenes
- Alkadienes
  - Cumulated alkadienes (1,2-alkadienes)
  - Conjugated alkadienes (1,3-alkadienes)
  - Isolated alkadienes (1,4-alkadienes)
- Alkynes
- Arenes

HYDROCARBON Halogen derivatives

- Hydrocarbon Halogen Derivatives with Csp3-Hal Bonds
  - Alkyl halides
    - Alkyl fluorides
    - Alkyl chlorides
    - Alkyl bromides
    - Alkyl iodides

Allyl halides
Allyl fluorides
Allyl chlorides
Allyl bromides
Allyl iodides
Benzyl halides
Benzyl fluorides
Benzyl chlorides
Benzyl bromides
Benzyl iodides
Gem-Dihalides *
Gem-Trihalides **
* Belong formally to carbonyl derivatives
** Belong formally to carboxyl derivatives

Hydrocarbon halogen derivatives with Csp2-Hal bonds
Vinyl halides
Vinyl fluorides
Vinyl chlorides
Vinyl bromides
Vinyl iodides
Aryl halides
Aryl fluorides
Aryl chlorides
Aryl bromides
Aryl iodides

Hydrocarbon halogen derivatives with Csp-Hal bonds
Alkynyl halides
Alkynyl fluorides
Alkynyl chlorides
Alkynyl bromides
Alkynyl iodides

Hydrocarbon Hydroxyl and Thiol Derivatives

Hydrocarbon hydroxyl derivatives with Csp3-O(S) bonds
Alcohols
Primary alcohols
Secondary alcohols
Tertiary alcohols
Thiols
Alkylthiols
1,2-Diols
1,2-Dithiols
1,2-Aminoalcohols
1,2-Aminothiols
Ethers
Dialkylethers
Alkylarylethers
Diarylethers
Thioethers
Dialkylthioethers

        Alkylarylthioethers
        Diarylthioethers
    Hydroperoxides
    Peroxides
    Disulfides

Hydrocarbon Hydroxyl Derivatives with Csp2-O(S) Bonds
    Enols
    Thioenols
    Enolethers
    Thioenolethers
    Enediols
    Enethiodiols
    Phenols
    Thiophenols
    Diphenols
        1,2-Diphenols
        1,3-Diphenols
        1,4-Diphenols
    Dithiophenols
        1,2-Dithiophenols
        1,3-Dithiophenols
        1,4-Dithiophenols
    Aminophenols
        1,2-Aminophenols
        1,3-Aminophenols
        1,4-Aminophenols
    Aminothiophenols
        1,2-Aminothiophenols
        1,3-Aminothiophenols
        1,4-Aminothiophenols

Hydrocarbon Hydroxyl Derivatives with Csp-O(S) Bonds
    Alkynyl alcohols
    Alkynyl thiols

(THIO)Carbonyl Compounds and Their Derivatives

Carbonyl compounds: aldehydes and ketones
    Aldehydes
    Ketones
        Quinones

Thiocarbonyl compounds: thioaldehydes and thioketones
    Thioaldehydes
    Thioketones
        Thioquinones

(Thio)carbonyl compounds derivatives
    Carbonyl hydrates
    Hemiacetals/Hemiketals
    Acetals/Ketals
    Thioacetals/Thioketals

Imines
Hydrazones
Oximes
Oxime ethers
Semicarbazones
Thiosemicarbazones

Ketenes
Ketene acetal derivatives

(THIO)Carboxylic Acid Derivatives

Carboxylic acid derivatives
Carboxylic acids
Carboxylic acid salts
Carboxylic acid esters
Lactones
Carboxylic acid anhydrides
Acyl halides
Acyl fluorides
Acyl chlorides
Acyl bromides
Acyl iodides
Carboxylic acid amides
Carboxylic acid primary amides
Carboxylic acid secondary amides
Carboxylic acid tertiary amides
Lactams
Carboxylic acid imides
Carboxylic acid unsubstituted imides
Carboxylic acid substituted imides
Carboxylic acid hydrazides
Carboxylic acid azides
Hydroxamic acids
Imido esters
Imidoyl halides
Carboxylic acid amidines
Carboxylic acid amidrazones
Nitriles
Isonitriles
Acyl cyanides

α-Substituted carboxylic acids
α-Aminoacids
α-Hydroxyacids
α-Oxoacids
α,β-Unsaturated carboxylic acids

Orthocarboxylic acid derivatives
Carboxylic acid orthoesters
Carboxylic acid amide acetals

Thiocarboxylic acid derivatives
Thiocarboxylic acids

- Thiocarboxylic acid esters
  - Thiolactones
- Thiocarboxylic acid amides
  - Thiolactams
- Imidothioesters

(THIO)Carbonic Acid Derivatives

- Carbonic acid derivatives
  - Carbonic acid esters
    - Carbonic acid monoesters
    - Carbonic acid diesters
  - Carbonic acid ester halides
  - Ureas
  - Isoureas
  - Guanidines
  - Semicarbazides
  - Carbodiimides
  - Cyanates
  - Isocyanates

- Thiocarbonic acid derivatives
  - Thiocarbonic acid esters
    - Thiocarbonic acid monoesters
    - Thiocarbonic acid diesters
  - Thiocarbonic acid ester halides
  - Thioureas
  - Isothioureas
  - Thiosemicarbazides
  - Isothiocyanates
  - Thiocyanates

(*THIO)Carbamic acid derivatives*

- Carbamic acid derivatives
  - Carbamic acid
  - Carbamic acid esters (urethanes)
  - Carbamic acid halides

- Thiocarbamic acid derivatives
  - Thiocarbamic acid esters
  - Thiocarbamic acid halides

Organonitrogen compounds

- Amines
  - Primary amines
    - Primary aliphatic amines
    - Primary aromatic amines
  - Secondary amines
    - Secondary aliphatic amines
    - Secondary mixed amines (aryl alkyl)
    - Secondary aromatic amines
  - Tertiary amines

Tertiary aliphatic amines
Tertiary mixed amines (aryl alkyl)
Tertiary aromatic amines
Quaternary ammonium salts
1,2-Diamines
1,2-Aminoalcohols
1,2-Aminothiols
Aminals
Hemiaminals
Thiohemiaminals

Aminophenols
1,2–Aminophenols
1,3–Aminophenols
1,4–Aminophenols
Aminothiophenols
1,2–Aminothiophenols
1,3–Aminothiophenols
1,4–Aminothiophenols

Enamines
Hydroxylamines
Hydrazine derivatives
Azo compounds
Azides
Diazonium salts
Nitroso compounds
Nitro compounds
N-Oxides
Nitrites
Nitrates

(Organo)sulfur COMPOUNDS

Sulfur (VI) Compounds

Sulfuric acid derivatives
Sulfuric acid
Sulfuric acid esters
Sulfuric acid monoesters
Sulfuric acid diesters
Sulfuric acid amide esters
Sulfuric acid amides
Sulfuric acid diamides

Sulfuryl halides

Sulfonic acid derivatives
Sulfonic acids
Sulfonic acid esters
Sulfonamides
Sulfonyl halides

Sulfones

Sulfur (IV) Compounds

Sulfinic acid derivatives

Sulfinic acids
Sulfinic acid esters
Sulfinic acid halides
Sulfinic acid amides

Sulfoxides

Sulfur (II) Compounds

Sulfenic acid derivatives
Sulfenic acids
Sulfenic acid esters
Sulfenic acid halides
Sulfenic acid amides

(Organo)phosphorus Compounds

Phosphorus (V) compounds

Phosphoric acid derivatives
Phosphoric acid
Phosphoric acid esters
Phosphoric acid halides
Phosphoric acid amides

Phosphonic acid derivatives
Phosphonic acids
Phosphonic acid esters
Phosphonic acid amides

Phosphinoxides

Thiophosphoric acid derivatives
Thiophosphoric acids
Thiophosphoric acid esters
Thiophosphoric acid halides
Thiophosphoric acid amides

Phosphorus (III) compounds

Phosphines

Organoboron Compounds

Boronic acids
Boronic acid esters

Organometallic Compounds

Organolithium compounds
Organomagnesium compounds
Other organometallic compounds

Heterocyclic Compounds

Any heterocyclic ring
Aromatic heterocyclic compounds
Five-membered heterocycles
Six-membered heterocycles
Oxohetarenes

- Thioxohetarenes
- Iminohetarenes

Three-membered heterocycles (LS)
- Three-membered heterocycles with one heteroatom (LS)
  - Saturated three-membered heterocycles with one heteroatom (LS)
  - Unsaturated three-membered heterocycles with one heteroatom (LS)
- Three-membered heterocycles with two heteroatoms (LS)
  - Saturated three-membered heterocycles with two heteroatoms (LS)
  - Unsaturated three-membered heterocycles with two heteroatoms (LS)
- Three-membered heterocycles with three heteroatoms (LS)
  - Saturated three-membered heterocycles with three heteroatoms (LS)
  - Unsaturated three-membered heterocycles with three heteroatoms (LS)

Three-membered heterocycles (HS)
- Three-membered heterocycles with one heteroatom (HS)
  - Saturated three-membered heterocycles with one heteroatom (HS)
    - Aziridines (HS)
    - Oxiranes (HS)
    - Thiiranes (HS)
  - Unsaturated three-membered heterocycles with one heteroatom (HS)
    - Azirines (HS)
    - 1H-Azirines (HS)
    - 2H-Azirines (HS)
    - Oxirenes (HS)
    - Thiirenes (HS)
- Three-membered heterocycles with two heteroatoms (HS)
  - Saturated three-membered heterocycles with two heteroatoms (HS)
    - Diaziridines (HS)
    - Dioxiranes (HS)
    - Oxaziridines (HS)
    - Thiaziridines (HS)
  - Unsaturated three-membered heterocycles with two heteroatoms (HS)
    - Diazirenes (HS)
      - 1H-Diazirenes (HS)
      - 3H-Diazirenes (HS)
    - Oxazirenes (HS)
    - Thiazerenes (HS)
- Three-membered heterocycles with three heteroatoms (HS)
  - Saturated three-membered heterocycles with three heteroatoms (HS)
    - Triaziridines (HS)
    - Trioxiranes (HS)
    - Trithiiranes (HS)
    - Oxadiaziridines (HS)
    - Dioxaziridines (HS)
    - Thiodiaziridines (HS)
    - Dithiaziridines (HS)
  - Unsaturated three-membered heterocycles with three heteroatoms (HS)
    - 1H-Triazirene (HS)
    - Oxadiazirenes (HS)
    - Thiodiazirenes (HS)

Four-membered heterocycles (HS)
- Four-membered heterocycles with one heteroatom (LS)

- Unsaturated four-membered heterocycles with one heteroatom (LS)
- Saturated four-membered heterocycles with one heteroatom (LS)

Four-membered heterocycles with two heteroatoms (LS)
- Saturated four-membered heterocycles with two heteroatoms (LS)
- Unsaturated four-membered heterocycles with two heteroatoms (LS)

Four-membered heterocycles with three heteroatoms (LS)
- Saturated four-membered heterocycles with three heteroatoms (LS)
- Unsaturated four-membered heterocycles with three heteroatoms (LS)

Four-membered heterocycles with four heteroatoms (LS)
- Saturated four-membered heterocycles with four heteroatoms (LS)
- Unsaturated four-membered heterocycles with four heteroatoms (LS)

Four-membered heterocycles with one heteroatom (HS)
- Saturated four-membered heterocycles with one heteroatom (HS)
  - Azetidines (HS)
  - Oxetanes (HS)
  - Thietanes (HS)
- Unsaturated four-membered heterocycles with one heteroatom (HS)
  - Azetines (HS)
    - 1-Azetines (HS)
    - 2-Azetines (HS)
  - Azetes (HS)
  - Oxetenes (HS)
  - Thietenes (HS)

Four-membered heterocycles with two heteroatoms (HS)
- Saturated four-membered heterocycles with two heteroatoms (HS)
  - Diazetidines (HS)
    - 1,2-Diazetidines (HS)
    - 1,3-Diazetidines (HS)
  - Dioxetanes (HS)
    - 1,2-Dioxetanes (HS)
    - 1,3-Dioxetanes (HS)
  - Dithietanes (HS)
    - 1,2-Dithietanes (HS)
    - 1,3-Dithietanes (HS)
- Unsaturated four-membered heterocycles with two heteroatoms (HS)
  - Diazetines (HS)
  - Dioxetenes (HS)
  - Dithietenes (HS)

Five-membered heterocycles (LS)
- Five-membered heterocycles with one heteroatom (LS)
  - Saturated five-membered heterocycles with one heteroatom (LS)
  - Unsaturated five-membered heterocycles with one heteroatom (LS)
  - Aromatic five-membered heterocycles with one heteroatom (LS)
- Five-membered heterocycles with two heteroatoms (LS)
  - Saturated five-membered heterocycles with two heteroatoms (LS)
  - Unsaturated five-membered heterocycles with two heteroatoms (LS)
  - Aromatic five-membered heterocycles with two heteroatoms (LS)
- Five-membered heterocycles with three heteroatoms (LS)
  - Saturated five-membered heterocycles with three heteroatoms (LS)
  - Unsaturated five-membered heterocycles with three heteroatoms (LS)
  - Aromatic five-membered heterocycles with three heteroatoms (LS)
- Five-membered heterocycles with four heteroatoms (LS)

Saturated five-membered heterocycles with four heteroatoms (LS)
Unsaturated five-membered heterocycles with four heteroatoms (LS)
Aromatic five-membered heterocycles with four heteroatoms (LS)
Five-membered heterocycles with five heteroatoms (LS)
Saturated five-membered heterocycles with five heteroatoms (LS)
Unsaturated five-membered heterocycles with five heteroatoms (LS)
Aromatic five-membered heterocycles with five heteroatoms (LS)

Five-membered heterocycles (HS)
Five-membered heterocycles with one heteroatom (HS)
Saturated five-membered heterocycles with one heteroatom (HS)
Pyrrolidines (HS)
Tetrahydrofurans (HS)
Tetrahydrothiophenes (HS)
Unsaturated five-membered heterocycles with one heteroatom (HS)
Pyrrolines (HS)
1-Pyrrolines (HS)
2-Pyrrolines (HS)
3-Pyrrolines (HS)
Dihydrofurans (HS)
2,3-Dihydrofurans (HS)
2,5-Dihydrofurans (HS)
Dihydrothiophenes (HS)
2,3-Dihydrothiophenes (HS)
2,5-Dihydrothiophenes (HS)
Aromatic five-membered heterocycles with one heteroatom (HS)
Pyrroles (HS)
Furans (HS)
Thiophenes (HS)
Indoles
Benzofurans
Benzothiophenes
Five-membered heterocycles with two heteroatoms (HS)
Saturated five-membered heterocycles with two heteroatoms (HS)
Diazolidines (HS)
Pyrazolidines (HS)
Imidazolidines (HS)
Dioxolanes (HS)
1,2-Dioxolanes (HS)
1,3-Dioxolanes (HS)
Dithiolanes (HS)
1,2-Dithiolanes (HS)
1,3-Dithiolanes (HS)
Unsaturated five-membered heterocycles with two heteroatoms (HS)
Pyrazolines (HS)
1-Pyrazolines (HS)
2-Pyrazolines (HS)
Imidazolines (HS)
2-Imidazolines (HS)
3-Imidazolines (HS)
4-Imidazolines (HS)
Dioxolenes (HS)
1,2-Dioxolenes (HS)

1,3-Dioxolenes (HS)
Dithiolenes (HS)
1,2-Dithiolenes (HS)
1,3-Dithiolenes (HS)
Aromatic five-membered heterocycles with two heteroatoms (HS)
Diazoles (HS)
Pyrazoles (HS)
Imidazoles (HS)
Oxazoles (HS)
1,2-Oxazoles (HS)
1,3-Oxazoles (HS)
Thiazoles (HS)
1,2-Thiazoles (HS)
1,3-Thiazoles (HS)
Five-membered heterocycles with three heteroatoms (HS)
Saturated five-membered heterocycles with three heteroatoms (HS)
Triazolidines (HS)
1,2,3-Triazolidines (HS)
1,2,4-Triazolidines (HS)
Trioxolanes (HS)
1,2,3-Trioxolanes (HS)
1,2,4-Trioxolanes (HS)
Trithiolanes (HS)
1,2,3-Trithiolanes (HS)
1,2,4-Trithiolanes (HS)
Unsaturated five-membered heterocycles with three heteroatoms (HS)
1,2,3-Triazolines (HS)
1,2,4-Triazolines (HS)
1,2,3-Trioxoles (HS)
1,2,3-Trithioles (HS)
Aromatic five-membered heterocycles with three heteroatoms (HS)
Triazoles (HS)
1,2,3-Triazoles (HS)
1,2,4-Triazoles (HS)
Oxadiazoles (HS)
1,2,3-Oxadiazoles (HS)
1,2,4-Oxadiazoles (HS)
1,2,5-Oxadiazoles (furazanes) (HS)
1,3,4-Oxadiazoles (HS)
Five-membered heterocycles with four heteroatoms (HS)
Saturated five-membered heterocycles with four heteroatoms (HS)
Tetrazolidines (HS)
Tetraoxolanes (HS)
Tetrathiolanes (HS)
Unsaturated five-membered heterocycles with four heteroatoms (HS)
Aromatic five-membered heterocycles with four heteroatoms (HS)
Tetrazoles (HS)
Oxatriazoles (HS)
1,2,3,4-Oxatriazoles (HS)
1,2,3,5-Oxatriazoles (HS)
Thiatriazoles (HS)
1,2,3,4-Thiatriazoles (HS)
1,2,3,5-Thiatriazoles (HS)

- Five-membered heterocycles with five heteroatoms (HS)
  - Saturated five-membered heterocycles with five heteroatoms (HS)
    - Pentazolidines (HS)
    - Pentaoxolanes (HS)
    - Pentathiolanes (HS)
  - Unsaturated five-membered heterocycles with five heteroatoms (HS)
  - Aromatic five-membered heterocycles with five heteroatoms (HS)
    - Pentazoles

Six-membered heterocycles (LS)
- Six-membered heterocycles with one heteroatom (LS)
  - Saturated six-membered heterocycles with one heteroatom (LS)
  - Unsaturated six-membered heterocycles with one heteroatom (LS)
  - Aromatic six-membered heterocycles with one heteroatom (LS)
- Six-membered heterocycles with two heteroatoms (LS)
  - Saturated six-membered heterocycles with two heteroatoms (LS)
  - Unsaturated six-membered heterocycles with two heteroatoms (LS)
  - Aromatic six-membered heterocycles with two heteroatoms (LS)
- Six-membered heterocycles with three heteroatoms (LS)
  - Saturated six-membered heterocycles with three heteroatoms (LS)
  - Unsaturated six-membered heterocycles with three heteroatoms (LS)
  - Aromatic six-membered heterocycles with three heteroatoms (LS)
- Six-membered heterocycles with four heteroatoms (LS)
  - Saturated six-membered heterocycles with four heteroatoms (LS)
  - Unsaturated six-membered heterocycles with four heteroatoms (LS)
  - Aromatic six-membered heterocycles with four heteroatoms (LS)
- Six-membered heterocycles with five heteroatoms (LS)
  - Saturated six-membered heterocycles with five heteroatoms (LS)
  - Unsaturated six-membered heterocycles with five heteroatoms (LS)
  - Aromatic six-membered heterocycles with five heteroatoms (LS)
- Six-membered heterocycles with six heteroatoms (LS)
  - Saturated six-membered heterocycles with six heteroatoms (LS)
  - Unsaturated six-membered heterocycles with six heteroatoms (LS)
  - Aromatic six-membered heterocycles with six heteroatoms (LS)

Six-membered heterocycles (HS)
- Six-membered heterocycles with one heteroatom (HS)
  - Saturated six-membered heterocycles with one heteroatom (HS)
    - Piperidines (HS)
    - Tetrahydropyrans (HS)
    - Tetrahydrothiopyrans (HS)
  - Unsaturated six-membered heterocycles with one heteroatom (HS)
    - Tetrahydropyridines (HS)
    - Dihydropyridines (HS)
    - Pyrans (HS)
    - Thiopyrans (HS)
  - Aromatic six-membered heterocycles with one heteroatom (HS)
    - Pyridines (HS)
- Six-membered heterocycles with two heteroatoms (HS)
  - Saturated six-membered heterocycles with two heteroatoms (HS)
    - Hexahydrodiazines
    - Dioxanes
    - Dithianes
    - Morpholines

- Unsaturated six-membered heterocycles with two heteroatoms (HS)
  - Tetrahydrodiazines (HS)
  - Dihydrodiazines (HS)
  - Dioxines (HS)
  - Dithiines (HS)
- Aromatic six-membered heterocycles with two heteroatoms (HS)
  - Pyridazines (HS)
  - Pyrimidines (HS)
  - Pyrazines (HS)

Six-membered heterocycles with three heteroatoms (HS)

- Saturated six-membered heterocycles with three heteroatoms (HS)
  - Hexahydrotriazines (HS)
  - Trioxanes (HS)
  - Trithianes (HS)
- Unsaturated six-membered heterocycles with three heteroatoms (HS)
- Aromatic six-membered heterocycles with three heteroatoms (HS)
  - Triazines (HS)

Six-membered heterocycles with four heteroatoms (HS)

- Saturated six-membered heterocycles with four heteroatoms (HS)
  - Hexahydrotetrazines (HS)
  - Tetroxanes (HS)
  - Tetrathianes (HS)
- Unsaturated six-membered heterocycles with four heteroatoms (HS)
  - Dihydrotetrazines (HS)
  - Tetrahydrotetrazines (HS)
- Aromatic six-membered heterocycles with four heteroatoms (HS)
  - Tetrazines (HS)

Six-membered heterocycles with five heteroatoms (HS)

- Saturated six-membered heterocycles with five heteroatoms (HS)
  - Hexahydropentazines (HS)
  - Pentoxanes (HS)
  - Pentathianes (HS)
- Unsaturated six-membered heterocycles with five heteroatoms (HS)
- Aromatic six-membered heterocycles with five heteroatoms (HS)
  - Pentazines

Six-membered heterocycles with six heteroatoms (HS)

- Saturated six-membered heterocycles with six heteroatoms (HS)
- Unsaturated six-membered heterocycles with six heteroatoms (HS)
- Aromatic six-membered heterocycles with six heteroatoms (HS)
  - Hexazines
